# Vitamin D and VDR in Gynecological Cancers—A Systematic Review

**DOI:** 10.3390/ijms18112328

**Published:** 2017-11-04

**Authors:** Eileen Deuster, Udo Jeschke, Yao Ye, Sven Mahner, Bastian Czogalla

**Affiliations:** Department of Obstetrics and Gynecology, University Hospital, LMU Munich, Marchioninistr 15, Munich 81377, Germany; Eileen.Deuster@med.uni-muenchen.de (E.D.); Udo.Jeschke@med.uni-muenchen.de (U.J.); Yao.Ye@med.uni-muenchen.de (Y.Y.); Sven.Mahner@med.uni-muenchen.de (S.M.)

**Keywords:** vitamin D, vitamin D receptor, VDR, gynecological cancers, ovarian, endometrial, cervical, vulvar, vaginal, cancer

## Abstract

In recent years, a vast amount of studies have centered on the role of vitamin D in the pathogenesis of certain types of cancers such as breast, colorectal and lung cancer. Increasing evidence suggests that vitamin D and its receptor play a crucial role in the development of gynecological cancers. In this review, we systematically analyzed the effect of vitamin D and the vitamin D receptor on endometrial, ovarian, cervical, vulvar and vaginal cancer. Our literature research shows that vitamin D levels and vitamin-D-related pathways affect the risk of gynecological cancers. Numerous ecological studies give evidence on the inverse relationship between UVB exposure and gynecological cancer risk. However, epidemiologic research is still inconclusive for endometrial and ovarian cancer and insufficient for rarer types of gynecological cancers. The vitamin D receptor (VDR) is upregulated in all gynecological cancers, indicating its influence on cancer etiology. The VDR polymorphism FokI (rs2228570) seems to increase the risk of ovarian cancer. Other nuclear receptors, such as the RXR, also influence gynecological cancers. Although there is limited knowledge on the role of the VDR/RXR on the survival of endometrial, cervical, vulvar or vaginal cancer patients, some studies showed that both receptors influence survival. Therefore, we suggest that further studies should focus on the vitamin D- and its hetero dimer receptor RXR in gynecological cancers.

## 1. Introduction

In the last two decades, vitamin D and its receptor (VDR) have gained significance. The fat-soluble steroid and VDR are commonly known to play a crucial role in the calcium homeostasis and metabolism [1]. However, the attention on vitamin D and its receptor increased when it was shown to influence significant medical problems such as cardiovascular disease, diabetes, and cancer [2,3,4]. A vast amount of preclinical and epidemiologic studies have focused on the impact vitamin D has on disease progression and mortality of cancer. High circulating levels of vitamin D are associated with a reduced risk of developing certain cancer types (breast, colorectal, gastric, hematological, head and neck, kidney, lung, ovarian, pancreatic liver, prostate and skin cancer). It has been demonstrated that vitamin D inhibits proliferation and induces differentiation of carcinoma cells in vitro and in vivo.

**Figure 1:**
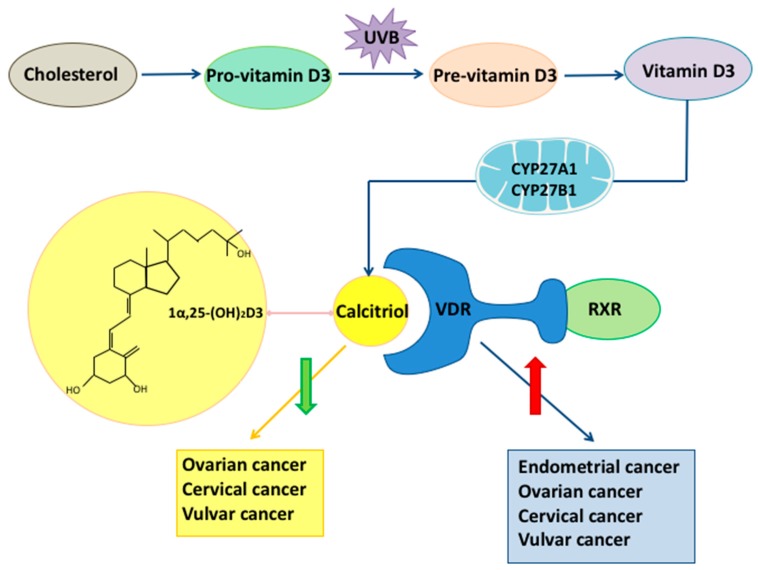
The role of vitamin D and vitamin D receptor (VDR) in gynecological cancers: Endogenous synthesis of vitamin D begins with the oxidation of cholesterol, resulting in pro-vitamin D3. In the skin, ultraviolet B (UVB) radiation transforms pro-vitamin D3 to pre-vitamin D3. Pre-vitamin D3 isomerizes to vitamin D3, also named as cholecalciferol. Two hydroxylations by the enzymes vitamin D 25-hydroxylases (CYP27A1) and renal mitochondrial 1-hydroxylase (CYP27B1) are necessary to transform vitamin D3 into the active 1α,25(OH)2D3. Different tissues, as well as gynecological cancer tissue, can synthesize calcitriol. 1α,25(OH)2D3 binds to the vitamin D receptor which belongs to the family of nuclear receptors and forms a complex with retinoid X receptor (RXR) to regulate gene expression. Both vitamin D and its receptor have a protective role in gynecological cancers. Low levels of vitamin D are found in ovarian, cervical and vulvar cancer. As a response to cancer, the expression of the vitamin D receptor is upregulated in endometrial, ovarian, cervical and vulvar cancer.

Vitamin D is obtained through two different ways (Figure 1): endogenous synthesis and diet; the endogenous synthesis being the primary source [5,6]. The synthesis of vitamin D is strictly regulated and dependent on UVB radiation. It starts in the bowel epithelium by the oxidation of cholesterol to 7-dehydrocholesterol, also known as pro-vitamin D3. Pro-vitamin D3 is transported to the skin and then converted to pre-vitamin D3 by ultraviolet radiation at wavelengths between 270–300 nm. In a temperature dependent reaction, pre-vitamin D3 isomerizes to cholecalciferol, vitamin D3. The activation of vitamin D3 to 1α,25(OH)2D3 (calcitriol) depends on two hydroxylations [7]. The first one takes place in the liver by the mitochondrial and microsomal vitamin D 25-hydroxylases (CYP27A1). Due to its physiological higher concentration, it is this which 25(OH)D (calcidiol) is commonly used in studies to evaluate the vitamin D level [8]. The second hydroxylation is carried out by the renal mitochondrial 1-hydroxylase (CYP27B1) [7,8]. The synthesis of calcitriol is not limited to renal cells, but can also be found in skin [9], prostate [10], keratinocytes [11] and cancer cells [12]. The active fat-soluble metabolite, 1α,25(OH)2D3, exercises his functions on different tissues by binding to the nuclear vitamin D receptor (VDR) [13].

The vitamin D receptor belongs to the superfamily of nuclear receptors. Its gene spans 75 kb and is located on the long arm of the chromosome 12 [14]. It interacts with other transcriptional factors; the best-studied one is the retinoid X receptor (RXR) [15] (Figure 1). By binding to genes with promoters containing a vitamin D response element (VDRE), the VDR receptor regulates gene expression. The VDR has been found in 30 different tissues. It has been shown that the role of VDR stretches beyond the calcium and vitamin d metabolism. Numerous in vivo and in vitro studies have demonstrated the importance of VDR as a mediator in inflammation, insulin-like growth factor signaling and estrogen-related pathways [16]. Furthermore, the VDR is expressed in a significant number of tumor tissues, indicating that the receptor influences cancer etiology. VDR polymorphisms have been demonstrated to change the activity of the vitamin D-VDR complex [17,18]. Their correlation to different cancer types has been investigated resulting in heterogeneous results. The most frequently polymorphisms associated with tumorigenesis are Bsm1, Fok1, Taq1, Apa1.

The interaction of VDR with the RXR implies that also the RXR may have a modulating effect on gynecological cancers. A recent study supports this thesis showing that the Retinoid X receptor and the VDR are overexpressed in BRCA1 mutated breast cancer cases, predicting the overall survival [19]. A high RXR expression was found in ovarian cancer cells [20]. Furthermore, it has been demonstrated that RXR polymorphisms play a role in the development of certain cancers [21,22]. An interaction between RXR and VDR polymorphisms was demonstrated, indicating their effects on the risk of ovarian cancer [23].

Considering that vitamin D and its receptor play a major role in the etiology of cancer, we reviewed the most updated evidence on their role in gynecologic cancers. Papers cited in this study came from the National Library of Medicine’s PubMed database (http://www.pubmed.gov). A systematic search was done for: “vitamin D” or “vitamin D receptor” in combination with “endometrial, ovarian, cervical, vulvar and vaginal cancer/tumor,” identifying 200 articles. In this review, we shed light on the role of vitamin D and VDR in endometrial, ovarian, cervical, vulvar and vaginal cancers.

## 2. Endometrial Cancer

Endometrial cancer is the most common gynecological malignant disease and the fifth most frequent cancer in women [19,20]. The five-year survival rate ranges from 74% to 91% in patients diagnosed in early stages [20]. The factors made responsible for developing endometrial cancer are older age, nulliparity, diabetes, estrogen-only hormone replacement therapy and obesity [21].

### 2.1. Vitamin D

Ecological studies gave evidence that UV exposure affects the risk of endometrial cancer by increasing vitamin D levels. Women living in higher altitudes have a higher risk than those living in lower altitudes. An inverse association between UVB irradiance and endometrial cancer incidence was demonstrated [22]. A Swedish study even suggested that the risk of endometrial cancer is reduced by 40% when women use sunbeds more than three times per year [23]. Epidemiologic research yielded heterogeneous results on the role of 1α,25(OH)2D3 in endometrial cancer [24]. A meta-analysis of three case-control studies concluded on no significant relation between intake of vitamin D and incidence of endometrial carcinoma [24]. In 2010, a study with 830 endometrial cancer cases measuring circulating concentrations of 25(OH)D supported this result [25]. Their findings did not indicate a protective role of vitamin D. Likewise, time-varying predicted plasma 25(OH)D was not found to be associated with endometrial cancer incidence [26].

Although vitamin D has been suggested not to affect endometrial cancer risk, it does suppress an obesity-induced increase of premalignant endometrial lesions in animal models. As stated above, obesity is an important risk factor for endometrial cancer. Yu et al., showed that dietary supplementation with vitamin D inhibits the carcinogenic effect of obesity on the endometrium [27].

### 2.2. Vitamin D Receptor (VDR)

The vitamin D receptor is expressed in non-pathological and pathological endometrial tissue [28]. The expression is independent of the menstrual cycle, not differing between the proliferative and secretory phase of the endometrium [29]. As anticipated, VDR levels in endometrial cancer are significantly higher than in control endometrium [28]. Studies examining the role of VDR in endometrial cancer are sparse.

## 3. Ovarian Cancer

Ovarian cancer is one of the five leading causes of cancer death among all ages in females [30]. Five-year survival rates are less than 45%. Its risk factors include gravidity, tubal ligation, premenopausal status, menopausal estrogens and contraceptive steroids [31].

### 3.1. Vitamin D

Ecological studies have demonstrated a lower incidence of ovarian cancer in southern countries, indicating a positive correlation between factors which inhibit vitamin D synthesis (e.g., latitude, sun exposure) and ovarian cancer risk [32].

Nevertheless, longitudinal studies on the role of circulating vitamin D in ovarian cancer have led to confounding results. In 2011, Yin et al. [33] published a meta-analysis of individual cohort studies examining the association between circulating vitamin D and ovarian cancer incidence. In this meta-analysis only four studies were left for inclusion, all of them (Tworoger et al. [34], Arslan et al. [35], Toriola et al. [36] and Zheng et al. [37]) reported no significant correlation between 25(OH)D and ovarian cancer risk. However, most of the studies found a tendency between low circulating 25(OH)D and ovarian cancer incidence. In 2016 a large mendelian randomization study was published [38]. 31,719 women of European ancestry were included. Results from this study suggest that genetically lowered 25(OH)D concentrations are associated with a higher incidence of ovarian cancer. This observation was reaffirmed by Anastasi et al. in a clinical study of 2016. They assessed the association between the Risk of Malignancy Algorithm (ROMA) and 25(OH)D levels in obese women. The ROMA score predicts the risk of developing epithelial ovarian cancer by combining the human epididymis protein 4 (HE4) and the CA 125 markers. High ROMA scores correlated with low 25(OH) levels [39].

Ovarian cancer patients have reduced 25(OH)D levels compared to the general population [40,41]. Higher 25-hydroxyvitamin D levels at diagnosis seem to be associated with longer survival among women with ovarian cancer [42]. However, Webb et al., indicated that 25(OH)D is not significantly related to progression-free survival.

Many studies have focused on the mechanistic pathway of vitamin D in ovarian cancer. 25 (OH)D inhibits proliferation and causes cell cycle arrest in ovarian carcinoma cells [43]. A primary target gene for 1α,25(OH)2D3 is GADD45, mediating G2/M transition [44]. Vitamin D’s synthetic analogous (EB1089) hinders in vitro growth and induces tumor suppression in animal models [45]. Recent findings have drawn attention to the role vitamin D plays in epithelial-mesenchymal-transition (EMT), a process being crucial for tumor progression. Hou et al. [46] showed that 1α,25(OH)2D3 reduces expression of transcription factors of EMT and thereby inhibits migration and invasion of SKOV-3 cells.

One of the reasons ovarian cancer has a high mortality is due to late diagnosis [31], resulting in an extended invasion of cancer in peritoneal organs. Vitamin D and its receptor have been shown to suppress epithelial ovarian cancer invasion into the omentum [47].

### 3.2. Vitamin D Receptor

The vitamin D receptor is expressed in non-pathological ovarian epithelium, as well as in ovarian tumors. The receptor is essential for full ovarian function by affecting estrogen biosynthesis and mediating aromatase gene expression [48]. VDR-null mice have been demonstrated to exhibit gonadal insufficiency and low aromatase activity. Different studies have shown that VDR expression is increased in ovarian cancer [49,50,51], indicating an endogenous response to tumor progression. In 2010 Silvagno et al., analyzed the correlation between VDR expression and clinicopathological parameters, reporting on no significant association [52]. A cross-link between VDR and androgen receptor, an important stimulator of growth in human ovarian cancer cells, has been postulated. Dihydrotestosterone upregulates the expression of VDR and hence the activity of 1,25 (OH)D3, resulting in a growth inhibition of the human ovarian cancer cell line OVCAR-3 [53].

**Figure 2:**
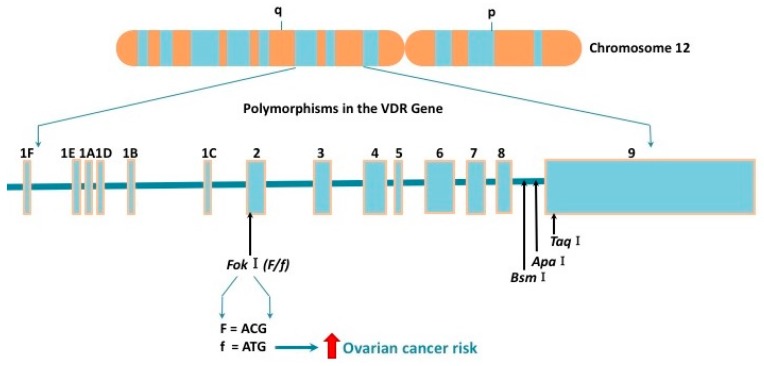
VDR polymorphisms in ovarian cancer: The VDR gene spans 75 kb of DNA and is located on the chromosome 12q12-14, consisting of six 5′ noncoding exons (1a–1f) and eight coding exons (2–9). Several VDR polymorphisms have been identified: Fok1, Bsm1, Apa1, and Taq1. The restriction fragment polymorphism of Fok1 alters an ACG codon resulting in an additional start codon and thereby generating a longer VDR protein. The f allele indicates the absence of the restriction site encoding a 427–amino acid protein; the F allele shows the presence of the restriction site encoding a 424–amino acid protein. However, the longer version of the VDR protein exerts less transcriptional activity. Carrying the ff or Ff genotypes increase the risk of ovarian cancer.

In recent years VDR polymorphisms gained much attention. Several polymorphs of VDR have been identified to decontrol vitamin D activity and thereby to affect its role in ovarian carcinogenesis (Figure 2). The most frequently studied single-nucleotide polymorphisms are two restriction fragment length polymorphisms: FokI (rs2228570) and BsmI (rs1544410). FokI has been indicated to increase the risk of ovarian cancer [54]. The f allele is a three amino acids longer version of the VDR protein having less transcriptional activity than the F allele. Carrying the FokI ff genotype increases ovarian cancer risk by 20% [54]. Likewise, the Ff genotype seems to correlate with a higher risk for ovarian cancer (Figure 2) [55]. It is well-established that VDR polymorphs vary depending on ethnicity [17]. The highest frequency of f allele is among Asians, followed by Caucasians and Africans [54]. Studies on the BsmI polymorphisms showed no association with ovarian cancer risk [55,56]. None of the genetic models for Apa1 and Taq1 supported an association with ovarian cancer risk [55].

## 4. Cervical Cancer

Cancer of the uterine cervix is the third most common gynecologic cancer and cause of death in the United States [30]. Global statistics have ranked the malignancy as the tenth most common cancer death in developed countries. However, the incidence gap between developed and developing countries is still wide. In countries which do not have access to cancer screening and prevention, cervical cancer remains a leading cause of cancer-related death, amounting to the second most common cancer in women. The overall five-year-survival rates from 2006 to 2012 were 68% [30]. The crucial risk factor for the malignancy is the human papillomavirus (HPV) [57]. Numerous studies have underlined the increased risk of cervical cancer caused by persistent HPV infections, immunodeficiencies and environmental factors (e.g., smoking) [57,58].

### 4.1. Vitamin D

In accordance with other types of gynecological cancers, ecological studies on vitamin D in cervical cancer have demonstrated an inverse correlation between solar ultraviolet B irradiance and cervical cancer incidence rate as well as mortality [59,60]. Other factors which might be related to cervical cancer risk do not confound this association [61]. A Japanese case-control study of 2010 was able to show a reduced risk of cervical neoplasia with growing vitamin D intake [62]. Recently, a clinical study focused on the effects long-term vitamin D supplementation has on the regression of cervical intraepithelial neoplasia grade 1 (CIN1). The double-blind, randomised clinical trial displayed a greater regression of CIN1 in women taking six months vitamin D supplementation than in the placebo group [63]. This effect might be possibly explained by the assumption that vitamin D deficiency can cause a persistent HPV infection and thus can lead to cervical pre-invasive lesions. Ozgu et al., showed that there is a statistically significant difference between 25(OH)D levels of HPV positive patients and the control group [64].

In vitro studies have suggested various mechanistic pathways in which vitamin D inhibits cervical cancer proliferation [65,66]. Among others, Wang et al., showed that vitamin D decreases the cervical cancer oncogene, HCCR-1, and increases p21 expression, thereby leading to cell cycle arrest at G1 [67]. Avila et al., displayed the inhibitory effect calcitriol has on human ether à-go-go-1 potassium channels (EAG1), which exhibit oncogenic properties [68]. In vivo studies have analyzed whether calcitriol increases the efficacy of radiation and could, therefore, be used as a potential therapy. However, F. Zhang et al., could not determine a significant benefit [69].

### 4.2. Vitamin D Receptor

In respect of the vitamin D receptor in cervical carcinoma, its expression is increased compared to non-pathological tissue [70]. It was shown that the VDR RNA-level and immunoreactivity is upregulated in cancer cells [49,71]. Friedrich, Meyberg, et al., analyzed the correlation of the VDR status with histopathological data such as tumor stage, tumor type, and lymph node status. They could not find a statistically significant association [72].

## 5. Vulvar Cancer

Vulvar cancer is the fourth most common gynecologic cancer, accounting for 3% to 5% of all genital cancers affecting women [30,73]. The five-year survival rate is 40% for patients with metastatic lymph nodes [74]. Vulvar cancer can be classified into two groups. The first one is associated with a human papillomavirus (HPV) infection occurring mostly in young women. The latter one is not related to HPV and presents itself in senior women.

### Vitamin D and VDR

Research elucidating the role of vitamin D and its receptor in vulvar cancer is still scarce. In 2012 the first study on the association between circulating 25(OH)D concentrations and vulvar cancer was published by Salehin et al. [75]. Serum 25(OH)D levels of 24 patients with vulvar cancer and 24 control patients were analyzed, resulting in no significant difference. Only for the under 50-year-old cancer group, a significant lower vitamin D level was detected. The same year another study focused on the VDR expression in vulvar cancer [76]. Higher levels of nuclear and cytoplasmatic VDR expression were found in pathological tissue compared to non-pathological tissue. The study could not show a significant correlation between VDR expression and overall survival.

The very limited number of studies analyzing vitamin D and its receptor in vulvar cancer impede drawing conclusions. Further studies with a larger number of patients need to focus on this topic. The same applies to research on the vitamin D receptor in vaginal cancer.

## 6. Vaginal Cancer

Vaginal carcinoma is a rare gynecological malignancy, constituting only 1–2% [77]. Five-year survival rate of vaginal cancer in early stages is 84% [78]. Risk factors for the malignancy are the number of sexual partners, history of cervical intraepithelial neoplasia, premalignant lesions in the vagina and HPV infection [79,80].

### Vitamin D and VDR

Vitamin D induces proliferation of vaginal epithelium [81]. Grant et al., suggested that disparities in vaginal cancer survival rates between African American and White American may be due to vitamin D [82]. 2004, a study was published reporting on the immunohistochemical detection of the vitamin D receptor in rat vaginal epithelium [83]. These results were confirmed in the human vagina by Kim et al., reporting on the presence of the VDR in all layers of the vaginal epithelium [84]. The expression of the vitamin D receptor is upregulated by vitamin D but seems not to correlate with the menstrual cycle. To our knowledge, the expression and role of the vitamin D receptor in vaginal carcinomas have yet not been analyzed. Further studies need to concentrate on this topic.

## 7. Conclusions

A large number of studies have displayed the crucial role vitamin D and its receptor have in gynecological cancers. Preclinical, as well as epidemiological evidence, supports vitamin D’s risk-reducing influence in gynecologic carcinomas (Figure 1). It is a widely shared opinion that vitamin D supplementation decreases the risk of developing cancer [85,86]. However, further randomized trials, need to ascertain this effect in gynecological cancers, taking into account the different serum levels of vitamin D. It would be worth considering whether vitamin D has an anti-oncogenic effect in all histopathological subtypes of the addressed cancers.

In the recent years, the vitamin D receptor has gained attention in gynecological cancers. VDR polymorphisms have been shown to affect the risk of ovarian cancer (Figure 2). More studies will need to focus on the VDR and its influence on endometrial, ovarian, cervical, vaginal and vulvar cancer. The relationship between vitamin D/VDR and gynecological cancers should be the focus of future studies which could lead to a better understanding of the molecular pathways. Furthermore, the interaction between the VDR and other nuclear receptors among others the estrogen receptor, the progesterone receptor and the androgen receptor should be further analyzed.

Nevertheless, the evidence reviewed in this paper indicates a key role of vitamin D and its receptor in gynecological cancers.

## Abbreviations

MDPI	Multidisciplinary Digital Publishing Institute
VDR	Vitamin D Receptor
UVB	Ultraviolet B
RXR	Retinoid X Receptor
HPV	Human Papillomavirus
